# Cryptic diversity in *Tranzscheliella* spp. (*Ustilaginales*) is driven by host switches

**DOI:** 10.1038/srep43549

**Published:** 2017-03-03

**Authors:** Ying-Ming Li, Roger G. Shivas, Lei Cai

**Affiliations:** 1 State Key Laboratory of Mycology, Institute of Microbiology, Chinese Academy of Sciences, Beijing, 100101, China; 2 College of Life Science, University of Chinese Academy of Sciences, Beijing, 100049, China; 3 Plant Pathology Herbarium, Department of Agriculture and Forestry, Dutton Park, Queensland, 4102, Australia

## Abstract

Species of *Tranzscheliella* have been reported as pathogens of more than 30 genera of grasses (Poaceae). In this study, a combined morphological and molecular phylogenetic approach was used to examine 33 specimens provisionally identified as belonging to the *T. hypodytes* species complex. The phylogenetic analysis resolved several well-supported clades that corresponded to known and novel species of *Tranzscheliella*. Four new species are described and illustrated. In addition, a new combination in *Tranzscheliella* is proposed for *Sorosporium reverdattoanum*. Cophylogenetic analyses assessed by distance-based and event-cost based methods, indicated host switches are likely the prominent force driving speciation in *Tranzscheliella*.

The genus *Tranzscheliella (Ustilaginales*) contains 17 species, which systematically infect the culms and inflorescences of about 33 genera of grasses (*Poaceae*) widely distributed around the world^1^,^2^. Lavrov^3^ first proposed the genus *Tranzscheliella* (type *T. otophora* on *Stipa pennata*, Turkmenistan) based on the presence of spores with two small bipolar cells, which were considered by Vánky^4^ to be circular broken parts of the thick exospore. Vánky^1^,^4^,^5^ broadened the concept of *Tranzscheliella* to include species with superficial, blackish brown sori that are either naked or have an ephemeral peridium on the culms or floral axis of grasses, and possess small (<8 μm diam.) spores. Molecular studies have shown that *Tranzscheliella* is monophyletic^6^,^7^.

With 165 grass species as hosts, *T. hypodytes s. lat*.^8^, represents a species complex that needs revision by modern molecular assessments^1^. Fischer and Hirschhorn^9^ noted more than 70 years ago that *T. hypodytes* (as *Ustilago hypodytes*) had for many years been applied to a complex of fungi, rather than a single species. The nomenclature and taxonomy of *T. hypodytes* has remained confused, with numerous synonyms as well as misidentified hosts reported in the scientific literature^1^.

Smut fungi are often host specific and host range is an important criterion for recognition of genera and species^6^,^10^, often supporting phylogenetic and biological studies^11^,^12^,^13^,^14^. Cospeciation was traditionally the main explanation for host-parasite cophylogenies^15^,^16^. With more available data and improved tools for cophylogenetic analyses, host switches rather than cospeciation, has become currently the most likely explanation for the diversification of many parasites, including fungal pathogens^17^,^18^. Host-shift speciation rather than cospeciation explained the cophylogenetic patterns of the smut fungus genus *Anthracoidea* found on species of the genus Carex (Cyperaceae)^19^.

Molecular phylogenetic methods have rarely been applied to *Tranzscheliella* spp. Further the cophylogenetic relationships between these smut fungi and their hosts are unknown. The aim of this study was to identify specimens that had been provisionally identified as *Tranzscheliella hypodytes*, mostly from China, using a combined morphological and molecular phylogenetic approach. This study resulted in the recognition of host specific species of *Tranzscheliella*, some of which are described here as new. Cophylogenetic analyses were used to determine the most likely explanation for speciation in *Tranzscheliella*.

## Results

**Figure 1:**
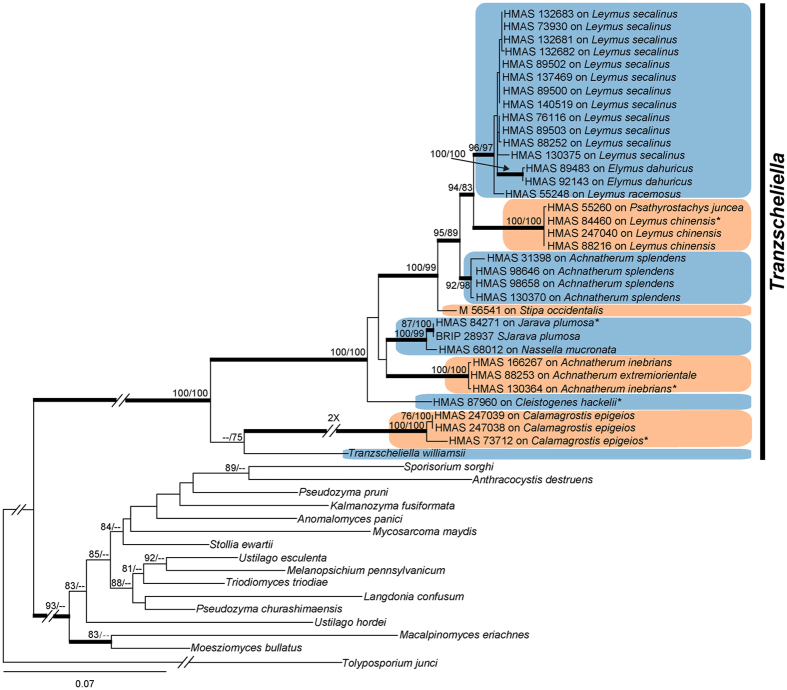
Phylogram obtained from a ML analysis based on the ITS and LSU sequence alignment. Values above the branches represent ML bootstrap values (>75%) from RaxML and PhyML analysis respectively. Thickened branches represent Bayesian posterior probabilities (>0.95). The scale bar indicates 0.07 expected substitutions per site. *Indicates type species.

**Figure 2:**
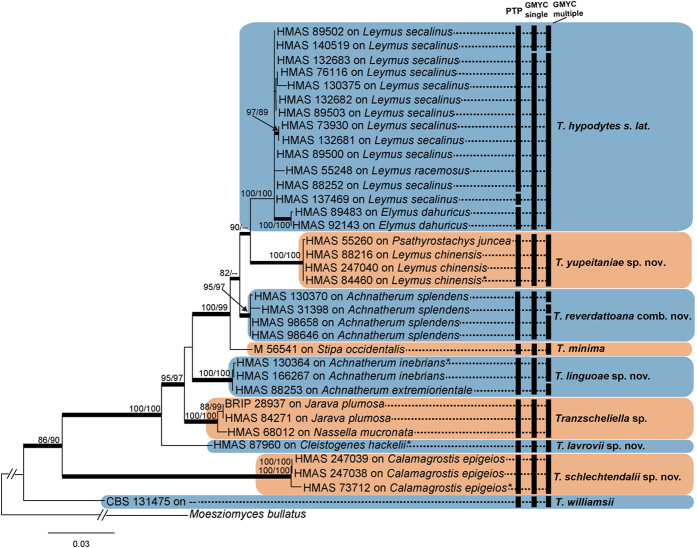
Phylogram obtained from a ML analysis based on the ITS and LSU sequence alignment. Values above the branches represent ML bootstrap values (>75%) from RaxML and PhyML analyses respectively. Thickened branches represent Bayesian posterior probabilities (>0.95). The scale bar indicates 0.03 expected substitutions per site. Asterisk indicates type species. The first column depicts species recognized by PTP model. The second and third columns depict putative species recognized by the single-threshold and multiple-threshold GMYC model, respectively.

The GenBank accession numbers of new sequences derived from this study, along with reference sequences, are showed in the Table 1. The sequences of the combined internal transcribed spacer (ITS) region of the rRNA gene and the large subunit (LSU) rRNA gene were aligned separately with gaps treated as missing characters. The evolutionary relationships of these sequences were analysed by maximum likelihood (ML) analyses and Bayesian probabilities. The inferred phylogenetic trees were consistent with each other, and only the PhyML tree is shown (Figs 1 and 2). *Tranzscheliella* spp. formed a well-supported monophyletic clade in the Ustilaginaceae (Fig. 1). Thirty-three specimens provisionally identified as belonging to the *T. hypodytes* species clustered in the seven well-supported clades (Fig. 2).

Thirty-five haplotypes of specimens provisionally identified as belonging to the *T. hypodytes* species complex, one as *T. minima* and one as *T. williamsii*, were used for coalescent analyses. The single-threshold general mixed Yule coalescent (GMYC) supported ten putative species, but this species delimitation scenario was not well supported by the likelihood ratio (LR) test (single-threshold: LR = 5.670471, *P* = 0.0587047). The multiple-threshold GMYC model provided a better fit to the ultrametric tree than a null model of uniform coalescent branching across the entire tree (multiple-threshold: LR = 7.168903, *P* = 0.02775189), which supported the delimitation of the taxa into thirteen putative species. The species delimitation results from GMYC and PTP analyses are summarized in Fig. 2. There was a high congruence between the PTP and multi-loci phylogenetic analyses. Both PTP and multiple-threshold GMYC analyses recovered six clades. Two clades formed single PTP groups, but multiple-threshold analysis separated each of these clades into two to three subclades. Another clade was recovered as a single group by phylogenetic analyses, but multiple-threshold GMYC and PTP analyses split this clade into two and three subclades respectively (Fig. 2). Based on concordant results from GMYC, PTP models and phylogenetic analyses, nine strongly supported clades were resolved, which represented four new species, a new combination, *T. minima*, a reduced *T. hypodytes s. lat*., and an unidentified *Tranzscheliella* sp. from South America. The pairwise identity of ITS sequences derived from the type of each species is shown in Table 2.

### Cophylogeny analysis

**Figure 3:**
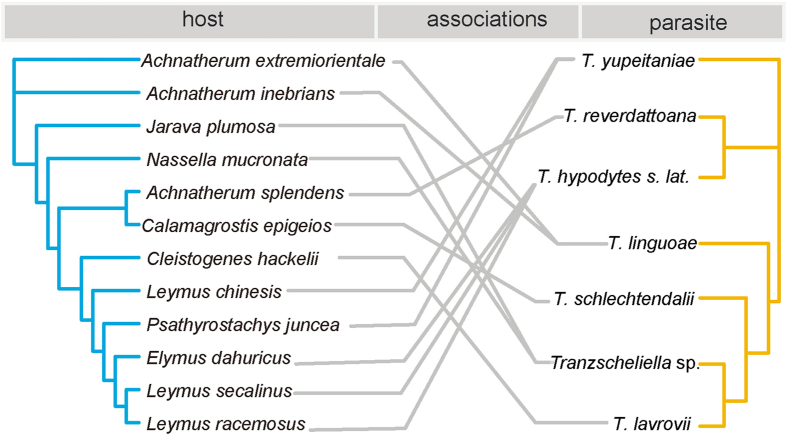
The tanglegram between *Tranzscheliella* species and their hosts. Fungal (right) and host grass (left) phylogenies from BI were used to generate the tanglegram using TreeMap 3.0ß.

The co-evolutionary relationships of the host and fungi are shown in Fig. 3. The global ParaFit test indicated that congruence between the phylogenies of *Tranzscheliella* species and their hosts was not significant (*P* = 0.50505) (Table 3). This indicated that co-speciation was not the major evolutionary force driving pathogen diversity and distribution on hosts. For the event based approach, all the reconstructions under different cost regimes were significantly better than those generated in the randomized test. Although different cost values were assigned to duplication, loss/sorting and failure to diverge, the event number inferred from analyses remained constant (0–1 duplication, 5–6 loss/sorting and 5 failure to diverge). The lowest costs were yielded by cost regime four and six, which penalized cospeciation. These two reconstructions comprised 0 cospeciation, 0 duplication, 6 host switches, 6 loss and 5 failures to diverge (Table 4).

### Taxonomy

Schlechtendal^20^ first described *Caeoma hypodytes*, which was subsequently transferred to several genera, namely, *Ustilago, Erysibe, Uredo, Cintractia* and *Tranzscheliella*. Hirschhorn^21^ considered that *Ustilago hypodytes* was a *nomen dubium* and proposed a neotype (referring to it as a lectotype) on *Elymus arenarius* (the type host) collected in 1884 by P. Sydow near Berlin, Germany, which had the advantage of being widely distributed in Rabenhorst’s *Fungi Europea Exsiccata*, Ser. 2, no. 3201. This species was subsequently transferred to *T. hypodytes*^8^. The nomenclature and taxonomy of *T. hypodytes* is confused, with numerous synonyms as well as misidentified hosts reported in the scientific literature^1^. *Tranzscheliella hypodytes* has long been recognized as a species complex rather than a single species^9^.

**Figure 4:**
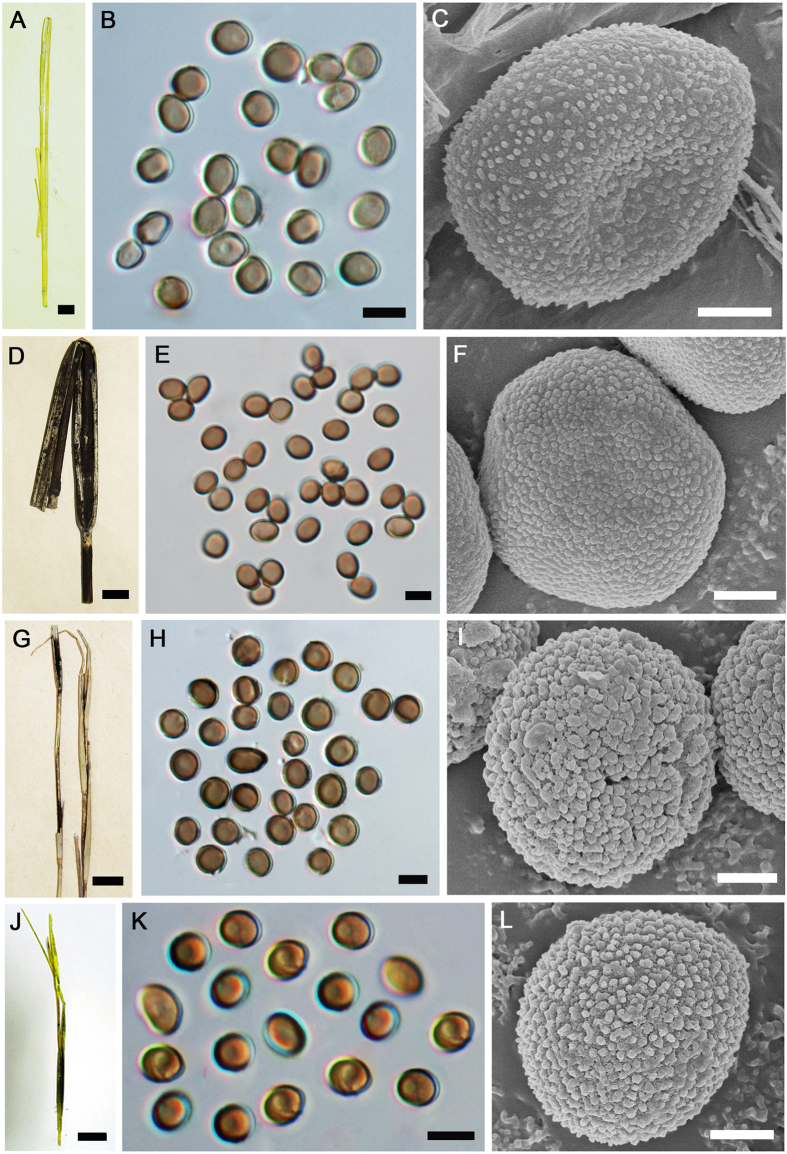
*Tranzscheliella hypodytes* (isoneotype HUV 3784) (**A**–**C**), *Tranzscheliella schlechtendalii* (HMAS 73712) (**D**–**F**), *Tranzscheliella lavrovii* (HMAS 87960) (**G**–**I**), and *Tranzscheliella* sp. (HMAS 84271) (**J**–**L**). **A**,**D**,**G**,**J**: Sori. **B,E,H,K**: Spores. **C,F,I,L**: Spores under SEM. *Bars*: **A,D,G,J**: 1 cm; **B,E,H,K**: 5 μm; **C,F,I,L**: 1 μm.

DNA could not be extracted from an isoneotype (HUV 3784) of *T. hypodytes*. Further, we were unable to obtain a more recent European specimen of *Tranzscheliella* on *Elymus arenarius*. Morphologically, *T. hypodytes* has spore walls that are smooth under light microscopy and densely, minutely, uniformly verruculose under SEM (p. 1007^1^; Fig. 4A–C), as compared to the denser and coarser warts seen under SEM in the taxa described here.

**Figure 5:**
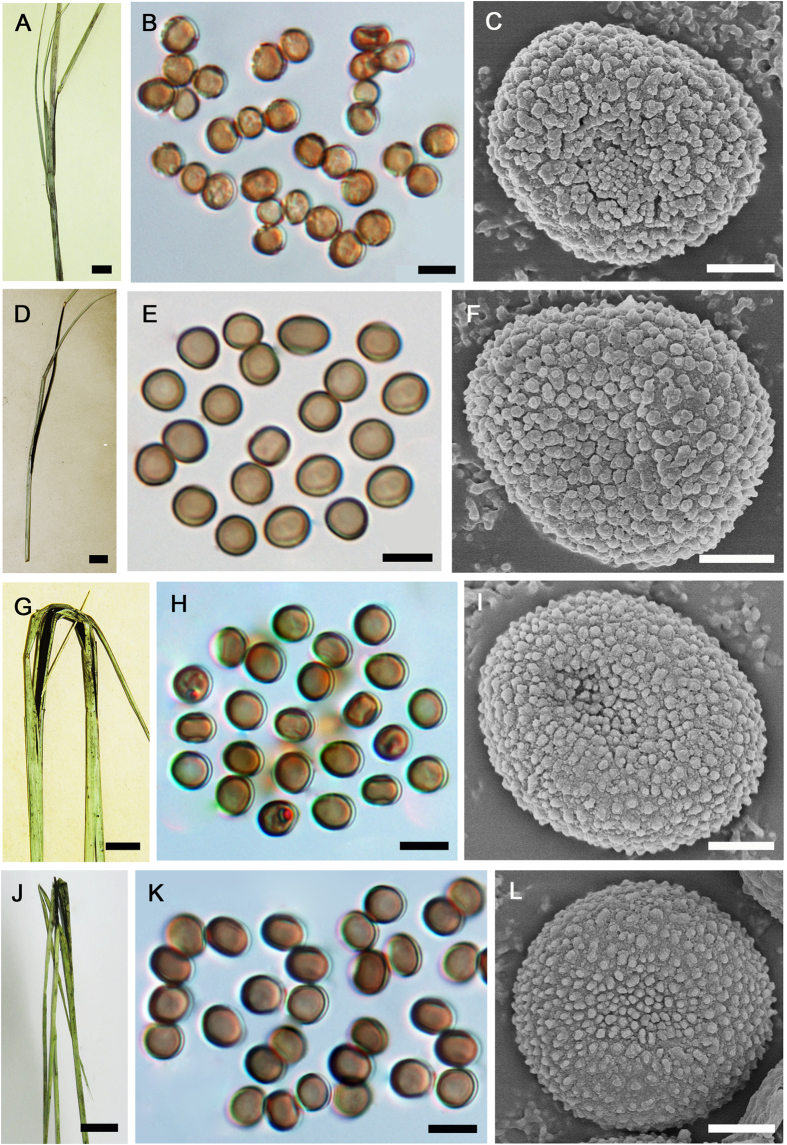
*Tranzscheliella linguoae* (HMAS 130364) (**A**–**C**), *Tranzscheliella yupeitaniae* (HMAS 84460) (**D**–**F**), *Tranzscheliella reverdattoana* (HMAS 98658) (**G**–**I**) and *Tranzscheliella hypodytes s. lat*. (HMAS 89483) (**J**–**L**). **A,D,G,J**: Sori. **B,E,H,K:** Spores. **C,F,I,L:** Spores. **C,F,I,L:** Spores under SEM. *Bars*: **A,D,G,J:** 1 cm; **B,E,H,K:** 5 μm; **C,F,I,L:** 1 μm.

***Tranzscheliella hypodytes*** (D.F.L. Schlechtendal) K. Vánky & E.H.C. McKenzie, *Smut Fungi of New Zealand*: 156, 2002, ***s**. **lat***. Fig. 5J–L.

Sori in the culms and surrounding the upper internodes and axes of abortive inflorescences, initially covered by the leaf sheath, finally exposed, peridium absent, upper internodes and leaves reduced in size. Spore mass semi-agglutinated to powdery. Spores globose, ovoid, ellipsoidal to slightly irregular, 4.5–5.5 × (3.5−) 4–4.5 (−5) μm, light olive-brown; wall c. 0.5 μm, surface smooth, in SEM moderately, unevenly verruculose, punctuate between warts.

Specimens examined: **China**, Inner Mongolia, Hohhot, on *Elymus dahuricus*, 7 Jul. 1961, S.J. Han, Q.M. Ma & R. Liu, HMAS 92143; Xinjiang, Emin, on *Leymus secalinus*, 2 Jun. 1985, Z.Y. Zhao, HMAS 73930; Xinjiang, Burqin, on *L. racemosus*, 2 Aug. 1986, Y.W. Xi, HMAS 55248; Ningxia, Zhongwei, on *L. secalinus*, 28 Aug. 1997, L. Guo, HMAS 76116; Qinghai, Ledu, on *L. secalinus*, 27 Sep. 2003, L. Guo & H.C. Zhang, HMAS 130375; Gansu, Wuwei, on *L. secalinus*, 27 Sep. 2003, L. Guo & H.C. Zhang, HMAS 89503; Gansu, Wuwei, on *L. secalinus*, 28 Sep. 2003, L. Guo & H.C. Zhang, HMAS 88252; Gansu, Shandan, on *Elymus dahuricus*, 2 Otc. 2003, L. Guo & H.C. Zhang, HMAS 89483; Gansu, Yuzhong, on *L. secalinus*, 10 Oct. 2003, H.C. Zhang, HMAS 89502; Gansu, Lanzhou, on *L. secalinus*, 12 Oct. 2003, H.C. Zhang, HMAS 89500; Qinghai, Ledu, on *L. secalinus*, 6 Aug. 2004, L. Guo & W. Li, HMAS 132683; Gansu, Wuwei, on *L. secalinus*, 12 Aug. 2004, L. Guo & W. Li, HMAS 140519; Qinghai: Gonghe, on *L. secalinus*, 12 Aug. 2004, L. Guo & W. Li, HMAS 132681; Qinghai, Ledu, on *Leymus secalinus*, 12 Aug. 2004, L. Guo & W. Li, HMAS 132682; Gansu, Lanzhou, on *L. secalinus*, 26 Jun. 2005, L. Guo, N. Liu & Z.Y. Li, HMAS 137469.

Note — The Chinese specimens of *Tranzscheliella* on *Elymus dahuricus* and *Leymus secalinus* (subfamily *Pooideae*, tribe *Triticeae*) formed an unresolved polytomy in the phylogenetic analysis (Fig. 2). There is a likelihood that this clade will contain *T. hypodytes s. str*., as the neotype was collected on *Leymus arenarius* from Germany in 1884^21^. Of note is that two specimens on *Elymus dahuricus*, which is native to Siberia, Mongolia and northern China, formed a strongly supported subclade that may represent a novel species. Taxonomic resolution of this polytomy needs to wait until the neotype of *T. hypodytes* has been sequenced and further specimens of *Tranzscheliella* on other triticoid grasses have been examined. Further, the spore morphology of the Chinese collections of *T. hypodytes* on species of *Elymus* and *Leymus* was similar to the type specimen of *T. hypodytes* on *L. arenarius* as compared with SEM images in Vánky^1^ (page 1007). Vánky^1^ listed several species of *Elymus* and *Leymus* as hosts of *T. hypodytes s. lat*., which is the classification that we assign to this clade.

***Tranzscheliella lavrovii***Y.M. Li, R.G. Shivas & L. Cai, **sp**. **nov**. Fig. 4G–I.

Fungal Name: FN570369.

Etymology: Named after Russian mycologist Nikolai Nicolaevich Lavrov, who established the genus *Tranzscheliella*.

Sori in the culms and surrounding the upper internodes and axes of abortive inflorescences, initially covered by the leaf sheath, finally exposed, peridium absent, upper internodes and leaves reduced in size. Spore mass semi-agglutinated to powdery. Spores globose, ovoid, ellipsoidal to slightly irregular, (4.5−) 5–6.5 (−7.5) × (4.5−) 5–6 μm, light olive-brown; wall c. 0.5 μm, surface smooth, in SEM densely verruculose.

Typification: **China**, Inner Mongolia, Xilin Gol Meng, on *Cleistogenes hackelii*, 14 Jul. 2003, L. Guo, W. Li & H.C. Zhang, HMAS 87960 (**holotype**).

Note — *Tranzscheliella lavrovii* occurs on *Cleistogenes hackelii* (subfamily *Chloridoideae*, tribe *Cynodonteae*), which has synonyms in *Diplachne* and *Kengia* that were considered as hosts for existing names in *Tranzscheliella*. Vánky^1^ lists *Diplachne* spp. as a host for four species of smut fungi, *T. amplexa, T. hypodytes s. lat*., *T. serena* and *U. ornata*. Of these, only *T. amplexa* and *T. hypodytes s. lat*., have small spores similar in size to *T. lavrovii*. However *T. lavrovii* has more densely verruculose spores in SEM than *T. amplexa*. In the phylogenetic analysis, *T. lavrovii* was distinct from other species studied, having ITS similarity ranging from 90–95% identity (Table 2). *Tranzscheliella lavrovii* has slightly larger spores than the isolates of *Tranzscheliella* sp. on *Stipa papposa* (4.5–5 × 4–4.5 μm).

***Tranzscheliella linguoae*** Y.M. Li, R.G. Shivas & L. Cai, **sp**. **nov**. Fig. 5A–C.

Fungal name: FN570370.

Etymology: Named after the Chinese mycologist Prof. Lin Guo, who specialises in the classification of Chinese smut fungi.

Sori in the culms and surrounding the upper internodes and axes of abortive inflorescences, initially covered by the leaf sheath, finally exposed, peridium absent, upper internodes and leaves reduced in size. Spore mass semi-agglutinated to powdery. Spores globose, ovoid, ellipsoidal to slightly irregular, 3.5–4 (−4.5) × 3–4 μm, light olive-brown; wall c. 0.5 μm, surface smooth, in SEM spore surface densely verruculose with irregular warts that fuse to create an irregular pattern on the spore surface.

Typification: **China**, Qinghai, Qilian, on *Achnatherum inebrians*, 2005, L. Guo & W. Li, HMAS 130364 (**holotype**).

Other specimens examined: **China**, Gansu, Tianzhu, on *A. extremiorientale*, 8 Oct. 2003, H.C. Zhang, HMAS 88253; Xinjiang, Urumqi, on *A. inebrians*, 23 Jul. 1959, Y.N. Yu, HMAS 166276.

Note —*Tranzscheliella linguoae* is one of four species of *Tranzscheliella* that infects species of *Achnatherum* (subfamily *Pooideae*, tribe *Stipeae*)^1^, which is another large polyphyletic grass genus^22^. The other species are *T. jacksonii, T. minima* and *T. williamsii*^1^. *Tranzscheliella linguoae* has smaller spores than *T. jacksonii* (8–13.5 × 8–12 μm) and *T. williamsii* (7–10 × 6–8 μm)^1^. The sori of *T. linguoae* lack a peridium and differ from *T. minima*, which has sori with a silvery to whitish fungal peridium^1^. In the phylogenetic analysis, specimens of *T. linguoae* were resolved in a well-supported monophyletic clade (Fig. 2).

***Tranzscheliella reverdattoana*** (Lavrov) Y.M. Li, R.G. Shivas & L. Cai, **comb**. **nov**. Fig. 5G–I.

Fungal name: FN570375.

Basionym: *Sorosporium reverdattoanum* Lavrov, *Trudy Tomsk. Gosud. Univ*. 86: 86. 1934.

Sori in the culms and surrounding the upper internodes and axes of abortive inflorescences, initially covered by the leaf sheath, finally exposed, peridium absent, upper internodes and leaves reduced in size. Spore mass semi-agglutinated to powdery. Spores globose, ovoid, ellipsoidal to slightly irregular, 4–4.5 (−5) × 3.5–4 (−4.5) μm, light olive-brown; wall c. 0.5 μm, surface smooth, in SEM densely verruculose and punctuate between warts.

Specimens examined: **China**, Xinjiang, Baicheng, on *Achnatherum* *splendens*, 22 Jul. 1959, J.H. Yu & Y.H. Yang, HMAS 31398; Gansu, Yumen, on *A. splendens*, 23 Aug. 2004, L. Guo & W. Li, HMAS 98658; Gansu, Yumen, on *A. splendens*, 23 Aug. 2004, L. Guo & W. Li, HMAS 98646; Gansu, Yumen, on *A. splendens*, 23 Aug. 2004, L. Guo & W. Li, HMAS 130370. **Kazakhstan**, Buran, Irtysh River, on *A. splendens*, 7 Jul. 1928, P.N. Golovin, HUV 12100 (**isotype** of *Sorosporium reverdattoanum*).

Note — *Sorosporium reverdattoanum* was described from a specimen of *Lasiagrostis splendens* (=*Achnatherum splendens*) (subfamily *Pooideae*, tribe *Stipeae*) collected in Kazakhstan^23^. Vánky^24^ observed that the spores of this specimen had passed through the alimentary tracts of insects, becoming agglutinated and hence the generic placement in *Sorosporium*. The host, *A. splendens*, is especially interesting as it was shown to form a highly supported monophyletic clade that was distinct from other Old World *Stipeae*^22^. Further, Hamasha *et al*.^22^ suggested that a new genus based on *A. splendens* was warranted, but only after clarification of the highly polyphyletic *Achnatherum*.

In making this new combination, we do not accept that *S. reverdattoanum* is a synonym of *T. minima* (type on *Achnatherum hymenoides*, USA) as considered by Vánky^1^,^24^. *Tranzscheliella reverdattoana* and *T. minima* both have very small spores (4–6 × 3.5–5 μm for *T. minima*) that are densely verruculose in SEM^1^. However *T. reverdattoana* has spore surfaces with punctate warts between the verrucose warts in SEM, which are not seen in *T. minima*^1^,^24^. There was sequence data on GenBank for a specimen identified as *T. minima* (DQ191251) on *Stipa occidentalis* (subfamily *Pooideae*, tribe *Stipeae*) from the USA, which was found to be sister to *T. reverdattoana* in our phylogenetic analysis (Fig. 2). Despite not having DNA sequence data from the type specimen of *S. reverdattoanum*, we have chosen to transfer this species to *Tranzscheliella* on the basis of the (i) similar morphology between the isotype of *S. reverdattoanum* and the Chinese specimens, (ii) relative proximity of the collections in neighboring countries, i.e. China and Kazakhstan, (iii) unique phylogenetic placement of *A. splendens* and (iv) molecular diversity between the North American isolate of *T. minima* (represented by DQ191251) and the Chinese isolates studied here.

***Tranzscheliella schlechtendalii*** Y.M. Li, R.G. Shivas & L. Cai, **sp**. **nov**. Fig. 4D–F.

Fungal Name: FN570371.

Etymology: Named after the great German botanist Diederich Franz Leonhard von Schlechtendal (1794–1866), who first described *Caeoma hypodytes*.

Sori in the culms and surrounding the upper internodes and axes of abortive inflorescences, initially covered by the leaf sheath, finally exposed, peridium absent, upper internodes and leaves reduced in size. Spore mass semi-agglutinated to powdery. Spores globose, ovoid, ellipsoidal to slightly irregular, (4.5−) 4.5–5.5 (−6) × (3.5−) 4–4.5 μm, light olive-brown; wall c. 0.5 μm, surface smooth, in SEM densely finely uniformly verruculose.

Typification: **China**, Inner Mongolia, Dengkou, on *Calamagrostis epigeios*, 4 Aug. 1996, Zhang & L. Guo, HMAS 73712 (**holotype**).

Other specimens examined: **China**, Gansu, 36° 12′ 41.7″N, 102° 02′ 63.4″, on *C. epigeios*, 4 Sep. 2013, Y.M. Li, R.G. Shivas, M.D.E. Shivas & Q. Chen, HMAS 247038; Gansu, 36° 12′ 41.7″N, 102° 02′ 63.4″, on *C. epigeios*, 4 Sep. 2013, Y.M. Li, R.G. Shivas, M.D.E. Shivas & Q. Chen, HMAS 247039.

Note — *Tranzscheliella schlechtendalii* is one of six species of smut fungi in the *Ustilaginaceae* that infect *Calamagrostis* (subfamily *Pooideae*, tribe *Poeae*), which is a large polyphyletic grass genus^25^. The other species include four *Ustilago* stripe smuts (*U. calamagrostidis, U. corcontica, U. scrobiculata* and *U. striiformis*)^26^ and *T. hypodytes s. lat*.^1^. The *Ustilago* stripe smuts all have larger spores that *T. schlechtendalii*. Vánky^1^ listed “? *Calamagrostis epigeios*” as a host of *T. hypodytes s. lat*., although a specimen was not found in Herbarium Ustilaginales Vánky. In the phylogenetic analysis, *T. schlechtendalii* was resolved on a long branch in a well-supported monophyletic clade that was sister to all other *Tranzscheliella* species except *T. williamsii* (Fig. 2).

***Tranzscheliella*** **sp**. Figure 4J–L.

Sori in the culms and surrounding the upper internodes and axes of abortive inflorescences, initially covered by the leaf sheath, finally exposed, peridium absent, upper internodes and leaves reduced in size. Spore mass semi-agglutinated to powdery. Spores globose, ovoid, ellipsoidal to slightly irregular, (4−) 4.5–5 (−5.5) × (3.5−) 4–4.5 (−5) μm, light olive-brown; wall c. 0.5 μm, surface smooth, in SEM densely verruculose.

Specimens examined: **Argentina**, 100 km NNE Bahia Blanca, on *Jarava plumosa (as Stipa papposa)*, 2 Dec. 1999, C. Vánky & K. Vánky, Vánky, Ust. Exs. 1110, HMAS 84271, BRIP 28937. **Ecuador**, on *Nassella mucronata*, 21 Mar. 1993, C. Vánky & K. Vánky, HUV 16016, HMAS 68012.

Note **—***Tranzscheliella* sp. occurs on two closely related grass species, *Jarava plumosa* and *Nassella mucronata*, (subfamily *Pooideae*, tribe *Stipeae*)^27^ in South America. Vánky^1^ listed three South American species, *Ustilago nummularia*^28^, *U. stipicola*^28^ and *U. spegazzinii*^29^, as synonyms of *T. hypodytes s. lat*., which may represent this species. In the phylogenetic analysis, *Tranzscheliella* sp. was resolved in a well-supported clade (Fig. 2). Further work is needed to determine the identity of this South American species.

***Tranzscheliella yupeitaniae*** Y.M. Li, R.G. Shivas & L. Cai, **sp**. **nov**. Fig. 5D–F.

Fungal Name: FN570377.

Etymology: Named after the Australian molecular biologist Yu Pei Tan, who collected this fungus with the authors in Inner Mongolia.

Sori in the culms and surrounding the upper internodes and axes of abortive inflorescences, initially covered by the leaf sheath, peridium absent, upper internodes and leaves reduced in size. Spore mass semi-agglutinated to powdery. Spores globose, ovoid, ellipsoidal to slightly irregular, 4–5 (−5.5) × 3–4 μm, light olive-brown; wall c. 0.5 μm, surface smooth, in SEM densely and irregularly verruculose.

Typification: **China**, Inner Mongolia, Barin Youqin, on *Leymus chinensis*, 31 Aug. 2001, L. Guo & H.C. Zhang, HMAS 84460 (**holotype**).

Other specimens examined: **China**, Xinjiang, Tacheng, on *Psathyrostachys juncea*, 10 Aug. 1986, Y.W. Xi, HMAS 55260; Inner Mongolia, Xilinhot, on *L. chinensis*, 17 Jul. 2003, L. Guo, W. Li & H.C. Zhang, HMAS 88126; Inner Mongolia, Xilinguole, on *L. chinensis*, 1 Jul. 2011, R.G. Shivas, M.D.E. Shivas, Y.P. Tan, Y. Zhang, L. Cai & Y.M. Li, BRIP 57343, HMAS 247040.

Note —*Tranzscheliella yupeitaniae* occurs on two closely related grass species, *Leymus chinensis* and *Psathyrostachys juncea* (subfamily *Pooideae*, tribe *Triticeae*)^30^,^31^. *Leymus* contains about 50 species found in temperate regions of China and North America, and *Psathyrostachys* about 10 species from Russia, Turkey and China^31^. Several species of *Leymus* were listed as hosts of *T. hypodytes s. lat*. by Vánky^1^. *Tranzscheliella yupeitaniae* has spores that are densely, unevenly verruculose in SEM, which differ from the densely, minutely, uniformly verruculose spores of *T. hypodytes s. str*.^1^ (p. 1007). In the phylogenetic analysis, *T. yupeitaniae* was resolved in a strongly supported clade (Fig. 2).

## Discussion

Many of the specimens examined were herbarium specimens more than 5 years old that had not been housed in environmentally controlled conditions. The extraction and amplification of DNA from these specimens was challenging, most likely because of DNA degradation. In term of genealogical information, the ITS and LSU (linked rDNA loci) equate to a single locus. GMYC and PTP are methods primarily intended for delimiting species in single-locus molecular phylogenies^32^,^33^, and the species boundaries proposed by these methods are consistent with the phylogenetic species concept^34^,^35^. The GMYC and PTP analyses used in this study meet the basic requirements of these two methods. The GMYC method has a tendency to over-split and generate biologically unrealistic putative entities^36^. In this study, *T. reverdattoana, T. schlechtendalii* and *Tranzscheliella* sp., formed single PTP groups, although multiple-threshold analysis separated each of these species into two subclades (Fig. 2). These subclades were not well supported by phylogeny, morphological characters and host affiliations. *Tranzscheliella schlechtendalii* was sister to all other *Tranzscheliella* spp., with a large molecular distance (ITS sequence identity 82–89%), indicating missing data or undiscovered species.

Traditional species recognition criteria for smut fungi have been based on morphological and ecological characters, with emphasis on sori, spores, sterile cells and columellae, as well as pathogenicity on specific hosts^1^,^13^. A high degree of host specificity in most smut fungi, as postulated by earlier mycologists, has been largely confirmed by phylogenetic studies^11^,^12^,^13^,^14^,^19^,^37^,^38^. In this study, phylogenetic analyses of specimens of *Tranzscheliella* recognized eight distinct species as well as a clade that we retain as representing *T. hypodytes s. lat*. These seven species, *T. lavrovii, T. linguoae, T. minima, T. reverdattoana, T. schlechtendalii, T. yupeitaniae* and *Tranzscheliella* sp., appear restricted to specific grass species or closely related grass species. The unidentified *Tranzscheliella* sp. was found on two closely related grass species, *Jarava plumosa* and *Nassella mucronata*, from South America. Most of the remaining specimens were collected from China (Gansu, Inner Mongolia, Ningxia, Xinjiang and Qinghai) and neighboring countries.

It is highly likely that more species of *Tranzscheliella* await discovery as only 13 grass host species were included in our study. Our data showed that specimens from the same host species in different geographical regions were genetically closer than the specimens from the same geographical region on different hosts. This indicates the importance of host-adaption in the process of speciation. Cophylogenetic analyses showed that host switch was the best explanation for speciation in *Tranzscheliella*.

## Materials and Methods

Specimens were borrowed from Queensland Plant Pathology Herbarium (BRIP) and Herbarium Mycologicum Academiae Sinicae (HMAS) (Table 1). Spores were mounted in lactic acid (100% v/v) and examined under the light microscope. Means and standard deviations (SD) were calculated from at least 20 measurements. Ranges were expressed as (min.−) mean − SD–mean + SD (−max.) with values rounded to 0.5 μm if below 20 μm and 1.0 μm if above 20 μm. Images were captured by using a Nikon Eclipse 80i camera attached to a Nikon DS-Fi1 compound microscope with Nomarski differential interference contrast. For scanning electron microscopy (SEM), dried spores were dusted onto double-sided adhesive tape, fixed on specimen stubs, sputter coated with gold, ca. 20 nm thick, and examined with a FEI Quanta 200 electron microscope. Nomenclatural novelties and descriptions were registered in MycoBank (www.MycoBank.org).

### DNA extraction, PCR amplification and sequencing

Fungal spores were removed from herbarium specimens with a fine needle and placed in cell lysis solution. For host tissue, dissected leaf samples were frozen in liquid nitrogen and ground with a mortar and pestle. Genomic DNA was extracted with the Gentra Puregene^®^ DNA Extraction Kit (Qiagen, Valencia, USA) according to the manufacturer’s instructions.

ITS was amplified with the primers M-ITS 1^11^ and ITS4^11^,^39^. LSU was amplified with the primers LR0R/LR5^40^. For the host plant, plasmid DNA regions *rbcL*, ITS and *trnH-psbA* were amplified with the primers rbcLa-F/rbcLa-R^41^,^42^, 17SE/26SE^43^ and psbAF/trnHR^44^, respectively. The PCR protocols were conducted as described by Zhang *et al*.^45^, with annealing temperature 62 °C for ITS of smuts, 60 °C for LSU, and 56 °C for ITS of host plants, *rbcL* and *trnH-psbA*. PCR products were sent to Biomed (Beijing, China) for sequencing with the same primer pairs used for amplification. Contigs were assembled in Mega 5^46^.

### Phylogenetic analyses

The DNA sequences included in this study (Table 1) were aligned online with MAFFT (mafft.cbrc.jp/alignment/server/index.html) (Katoh and Toh 2008) using the L-INS-i method. ML was implemented as a search criterion in RAxML^47^ and PhyML 3.0^48^. GTRGAMMA was specified as the model of evolution in both programs. The RAxML analyses were run with a rapid Bootstrap analysis (command -f a) using a random starting tree and 1,000 ML bootstrap replicates. The PhyML analyses were implemented using the ATGC bioinformatics platform (available at: http://www.atgcmontpellier.fr/phyml/), with six substitution type and SPR tree improvement, and support obtained from an approximate likelihood ratio test^49^.

MrBayes was used to conduct a Markov Chain Monte Carlo (MCMC) search in a Bayesian analysis. Four runs, each consisting of four chains, were implemented until the standard deviation of split frequencies were 0.02. The cold chain was heated at a temperature of 0.25. Substitution model parameters were sampled every 1,000 generations and trees were saved every 1,000 generations. Convergence of the Bayesian analysis was confirmed using AWTY^50^ (available at: ceb.csit.fsu.edu/awty/).

### Coalescent-based species delimitation

#### GMYC analysis

The combined ITS and LSU sequences were analysed under the single threshold model and the multi-threshold model. The alignments were stripped of non-unique haplotypes using Arlequin 3.1^51^. Haplotype alignments were used to generate gene trees using Beast 1.7.5 with an uncorrelated lognormal relaxed clock model^52^ and nucleotide substitution model using the same parameters as in the Bayesian analysis. Four independent MCMC chains were run for 400,000,000 generations, with sampling every 10,000 generations, using the ‘auto optimize’ operators option, and a Yule tree prior. The effective sample size (ESS) of each run was determined using Tracer v1.5 and only trees with an ESS of at least 200 were kept^53^. Four separate tree files were combined by LogCombiner^54^ (burnin = 40,000) with a reduced resample frequency of 200,000. The reduced tree samples were used to reconstruct the maximum clade credibility tree by TreeAnnotator^54^. The selected topologies were used to optimize the single-threshold and multi-threshold GMYC models online (http://species.h-its.org/gmyc/).

#### PTP analysis

The RAxML gene trees were constructed using the same markers selected by GMYC analysis. The PTP analysis was conducted online (http://species.h-its.org/ptp/) with the following settings: 10,000 MCMC generations; thinning interval of 100 and burn-in of 0.2^34^.

### Cospeciation analyses

TREEMAP 3b^55^ was used to generate a tanglegram from the ML tree of *Tranzscheliella* spp. and their host plants. To assess cospeciation between the host and parasites, both distance-based and event-based methods were utilized for the cophylogenetic analyses. For each of these two analyses, the parasite topology was obtained by using PhyML analysis based on ITS and LSU alignment, including just one representative per putative species. The host topology was obtained by PhyML analysis based on *rbcL*, ITS and *trnH-psbA* alignment of the representative specimens. For the distance-based analyses of cophylogeny, COPYCAT 2.02^56^ was used, which incorporated a wrapper for ParaFit^57^. The congruence between the host and parasites phylogenies were computed and statistical significance tests were assessed by comparing randomizing parasites and host association with 999 permutations^58^,^59^. Event-based analyses were run in Jane 4^60^.

Jane 4 considers five types of co-evolutionary event, namely cospeciation, duplication, host switch, sorting and failure to diverge. As it is difficult to estimate the relative cost of events, a default event cost scheme (cospeciation = 0, duplication = 1, duplication and host switch = 2, sorting = 1, failure to diverge = 1) as well as 9 cost regimes derived from default one were tested. In all the analyses, the vertex-base cost model method has been implemented, with the number of generation has been set to 100, and population size to 300. And the statistical significance of reconstructions was evaluated with 1,000 random tip mapping permutations.

## Additional Information

**How to cite this article:** Li, Y.-M. *et al*. Cryptic diversity in *Tranzscheliella* spp. (*Ustilaginales*) is driven by host switches. *Sci. Rep.* **7**, 43549; doi: 10.1038/srep43549 (2017).

**Publisher's note:** Springer Nature remains neutral with regard to jurisdictional claims in published maps and institutional affiliations.

## Figures and Tables

**Table 1:** List of species, herbarium accession numbers, hosts and GenBank accession numbers for specimens examined in this study.

Species	Herbarium no.^a^	Country	Host	GenBank accession no.	
				ITS	LSU
*Anomalomyces panici*	BRIP 46421	Australia	*Panicum trachyrachis*	DQ459348	DQ459347
*Anthracocystis destruens*	Ust. Exs. 472	Romania	*Panicum miliaceu*	AY344976	AY747077
*Dirkmeia churashimaensis*	OK 96	Japan	—	AB548947	AB548955
*Kalmanozyma fusiformata*	JCM 3931	Japan	—	AB089366	AB089367
*Langdonia confusum*	BRIP 42670	Australia	*Aristida queenslandica*	HQ013095	HQ013132
*Macalpinomyces eriachnes*	BRIP 39636	Australia	*Eriachne obtusa*	**KX686925**	**KX686955**
*Melanopsichium pennsylvanicum*	H.U.V. 17548	India	*Polygonum glabrum*	AY740040	AY740093
*Moesziomyces bullatus*	CBS 425.34	USA	*Paspalum distichum*	DQ831013	DQ831011
*Mycosarcoma maydis*	PBM 2469	USA	*Zea mays*	AY854090	AF453938
*Pseudozyma pruni*	BCRC 34227	Taiwan	*Prunus mume*	EU379942	EU379943
*Sporisorium sorghi*	MP 2036	Nicaragua	*Sorgum bicolor*	AY740021	AF009872
*Stollia ewartii*	BRIP 51818	Australia	*Sarga timorense*	HQ013087	HQ013127
*Tolyposporium junci*	H.U.V. 17169	Poland	*Juncus bufonius*	AY344994	AF009876
*Tranzscheliella hypodytes s. lat*.	HMAS 92143	China	*Elymus dahuricus*	**KX832829**	**KX832862**
*T. hypodytes s. lat*.	HMAS 132682	China	*Leymus secalinus*	**KX832833**	**KX832866**
*T. hypodytes s. lat*.	HMAS 89502	China	*Leymus secalinus*	**KX832832**	**KX832865**
*T. hypodytes s. lat*.	HMAS 137469	China	*Leymus secalinus*	**KX832836**	**KX832869**
*T. hypodytes s. lat*.	HMAS 89500	China	*Leymus secalinus*	**KX832828**	**KX832861**
*T. hypodytes s. lat*.	HMAS 140519	China	*Leymus secalinus*	**KX832830**	**KX832863**
*T. hypodytes s. lat*.	HMAS 130375	China	*Leymus secalinus*	**KX832838**	**KX832871**
*T. hypodytes s. lat*.	HMAS 89503	China	*Leymus secalinus*	**KX832835**	**KX832868**
*T. hypodytes s. lat*.	HMAS 76116	China	*Leymus secalinus*	**KX832827**	**KX832860**
*T. hypodytes s. lat*.	HMAS 88252	China	*Leymus secalinus*	**KX832831**	**KX832864**
*T. hypodytes s. lat*.	HMAS 73930	China	*Leymus secalinus*	**KX832826**	**KX832859**
*T. hypodytes s. lat*.	HMAS 132681	China	*Leymus secalinus*	**KX832837**	**KX832870**
*T. hypodytes s. lat*.	HMAS 132683	China	*Leymus racemosus*	**KX832834**	**KX832867**
*T. hypodytes s. lat*.	HMAS 89483	China	*Elymus dahuricus*	**KX832814**	**KX832847**
*T. lavrovii*	HMAS 87960^T^	China	*Cleistogenes hackelii*	**KX832843**	**KX832876**
*T. linguoae*	HMAS 166276	China	*Achnatherum extremiorientale*	**KX832820**	**KX832853**
*T. linguoae*	HMAS 88253	China	*Achnatherum inebrians*	**KX832818**	**KX832851**
*T. linguoae*	HMAS 130364^T^	China	*Achnatherum inebrians*	**KX832819**	**KX832852**
*T. minima*	M 56541	USA	*Stipa occidentalis*	**DQ191251**	**DQ191257**
*T. reverdattoana*	HMAS 55248	China	*Achnatherum splendens*	**KX832825**	**KX832858**
*T. reverdattoana*	HMAS 98658	China	*Achnatherum splendens*	**KX832823**	**KX832856**
*T. reverdattoana*	HMAS 98646	China	*Achnatherum splendens*	**KX832822**	**KX832855**
*T. reverdattoana*	HMAS 31398	China	*Achnatherum splendens*	**KX832821**	**KX832854**
*T. schlechtendalii*	HMAS 247039	China	*Calamagrostis epigeios*	**KX832844**	**KX832877**
*T. schlechtendalii*	HMAS 247038	China	*Calamagrostis epigeios*	**KX832845**	**KX832878**
*T. schlechtendalii*	HMAS 73712^T^	China	*Calamagrostis epigeios*	**KX832846**	**KX832879**
*T. williamsii*	CBS 131475	USA	—	JN367310	JN367338
*T. yupeitaniae*	HMAS 130370	China	*Psathyrostachys juncea*	**KX832824**	**KX832857**
*T. yupeitaniae*	HMAS 55260	China	*Leymus chinensis*	**KX832839**	**KX832872**
*T. yupeitaniae*	HMAS 88126	China	*Leymus chinesis*	**KX832841**	**KX832874**
*T. yupeitaniae*	HMAS 247040	China	*Leymus chinensis*	**KX832842**	**KX832875**
*T. yupeitaniae*	HMAS 84460^T^	China	*Leymus chinensis*	**KX832840**	**KX832873**
*Tranzscheliella* sp.	HMAS 84271	Argentina	*Jarava plumosa*	**KX832816**	**KX832849**
*Tranzscheliella* sp.	BRIP 28937	Argentina	*Jarava plumosa*	**KX832815**	**KX832848**
*Tranzscheliella* sp.	HMAS 68012	Ecuador	*Nassella mucronata*	**KX832817**	**KX832850**
*Triodiomyces triodiae*	BRIP 49124	Australia	*Triodia microstachya*	AY740074	AY740126
*Ustilago hordei*	Ust. Exs. 784	Iran	*Hordeum vulgare*	AY345003	AF453934
*Yenia esculenta*	Ust. Exs. 590	China	*Zizania latifolia*	AY345002	AF453937

Sequences generated in this study are shown in bold.

^a^Mycologicum; BCRC = Bioresource Collection and Research Center, Food Industry Research and Development Institute, Hsinchu, Taiwan; BRIP = Queensland Plant Pathology Herbarium, Dutton Park, Australia; CBS = CBS-KNAW Fungal Biodiversity Centre, Utrecht, Netherlands; HMAS = Herbarium Mycologicum Academiae Sinicae; H.U.V. = Herbarium Ustilaginales Vánky; MP = Herbarium Meike Piepenbring; M = Botanische Staatssammlung München, Germany; Ust. Exs. = Vánky, Ustilaginales exsiccata.

^T^Type specimen.

**Table 2:** The pairwise identity of the ITS sequences.

Identity of the ITS sequences	*T. schlechtendalii*	*T. lavrovii*	*Tranzscheliella* sp.	*T. linguoae*	*T. yupeitaniae*	*T. minima*	*T. reverdattoana*	*T. hypodytes s. lat.*
*T. williamsii*	82%	84%	84%	89%	84%	89%	90%	89%
*T. schlechtendalii*		88%	89%	89%	82%	82%	88%	88%
*T. lavrovii*			93%	93%	90%	93%	95%	91%
*Tranzscheliella* sp.				93%	91%	92%	94%	91%
*T. linguoae*					92%	94%	96%	94%
*T. yupeitaniae*						94%	95%	94%
*T. minima*							98–99%	95%
*T. reverdattoana*								97%

**Table 3:** Results of the cophylogenetic analyses with the distance-based approach ParaFit.

Parasite	Host	Total Links	*P*-value for global fit
Full dataset	12	12	0.50505
*T. schlechtendalii*	*Calamagrostis epigeios*	1	0.63636
*T. lavrovii*	*Cleistogenes hackelii*	1	0.05051
*Tranzscheliella* sp.	*Nassella mucronata*	1	0.38384
*Tranzscheliella* sp.	*Jarava plumosa*	1	0.92929
*T. linguoae*	*Achnatherum extremiorientale*	1	0.14141
*T. linguoae*	*Achnatherum inebrians*	1	0.25253
*T. yupeitaniae*	*Leymus chinesis*	1	0.34343
*T. yupeitaniae*	*Psathyrostachys juncea*	1	0.38384
*T. reverdattoana*	*Achnatherum splendens*	1	0.61616
*T. hypodytes s. lat*.	*Elymus dahuricus*	1	0.68687
*T. hypodytes s. lat*.	*Leymus secalinus*	1	0.63636
*T. hypodytes s. lat*.	*Leymus racemosus*	1	0.55556

**Table 4:** Results of the cophylogeny analyses using Jane 4.

Cost regime	Cost assigned to each event (C, D, HS, L, FD)	Event					Total cost	RTM-*P* value
		C	D	HS	L	FD		
1	0, 1, 2, 1, 1	3	0	3	6	5	17	0.011**
2	1, 1, 1, 1, 1	1	0	5	5	5	16	0.018**
3	1, 0, 0, 1, 1	0	0	6	6	5	11	0.013**
**4**	**1**, **0**, **0**, **1**, **0**	**0**	**0**	**6**	**6**	**5**	**6**	**0**.002**
5	2, 0, 1, 1, 0	0	0	6	6	5	12	0.023**
**6**	**2**, **0**, **0**, **1**, **0**	**0**	**0**	**6**	**6**	**5**	**6**	**0**.023**
7	2, 0, 1, 1, 1	0	0	6	6	5	17	0.019**
8	2, 0, 2, 1, 0	2	1	3	6	5	16	0.013**
9	2, 0, 2, 1, 1	2	1	3	6	5	21	0.014**
10	2, 0, 2, 2, 1	1–2	0	4–5	5	5	27	0.021**

Order of event cost is: C (cospeciation); D (duplication); HS (duplication & host switch); L (loss/sorting); FD (failure to diverge). Solutions of lowest overall cost are highlighted in bolds. The *P*-value of each randomized test using Random Tip Mapping (RTM) method was indicated, and Asterisk (*) indicate level of significanece of RTM.
